# Inline 3D Volumetric Measurement of Moisture Content in Rice Using Regression-Based ML of RF Tomographic Imaging

**DOI:** 10.3390/s22010405

**Published:** 2022-01-05

**Authors:** Abd Alazeez Almaleeh, Ammar Zakaria, Latifah Munirah Kamarudin, Mohd Hafiz Fazalul Rahiman, David Lorater Ndzi, Ismahadi Ismail

**Affiliations:** 1 Faculty of Electrical Engineering Technology, Universiti Malaysia Perlis (UniMAP), Arau 02600, Malaysia; almaleeh1987@yahoo.com (A.A.A.); hafiz@unimap.edu.my (M.H.F.R.); 2 Advanced Sensor Technology, Centre of Excellence (CEASTech), Universiti Malaysia Perlis (UniMAP), Arau 02600, Malaysia; latifahmunirah@unimap.edu.my; 3 Faculty of Electronic Engineering Technology, Universiti Malaysia Perlis (UniMAP), Arau 02600, Malaysia; 4 School of Computing, Engineering and Physical Sciences, University of the West of Scotland, Paisley PA1 2BE, UK; David.Ndzi@uws.ac.uk; 5 Space Science System, Sdn. Bhd., No. 17A, Tingkat 1, Jalan SPU 2, Saujana Business Park, Bandar Saujana Putra, Jenjarom 42600, Malaysia; ismahadi@s3sb.my

**Keywords:** 3D volumetric, moisture content, machine learning, tomographic imaging

## Abstract

The moisture content of stored rice is dependent on the surrounding and environmental factors which in turn affect the quality and economic value of the grains. Therefore, the moisture content of grains needs to be measured frequently to ensure that optimum conditions that preserve their quality are maintained. The current state of the art for moisture measurement of rice in a silo is based on grab sampling or relies on single rod sensors placed randomly into the grain. The sensors that are currently used are very localized and are, therefore, unable to provide continuous measurement of the moisture distribution in the silo. To the authors’ knowledge, there is no commercially available 3D volumetric measurement system for rice moisture content in a silo. Hence, this paper presents results of work carried out using low-cost wireless devices that can be placed around the silo to measure changes in the moisture content of rice. This paper proposes a novel technique based on radio frequency tomographic imaging using low-cost wireless devices and regression-based machine learning to provide contactless non-destructive 3D volumetric moisture content distribution in stored rice grain. This proposed technique can detect multiple levels of localized moisture distributions in the silo with accuracies greater than or equal to 83.7%, depending on the size and shape of the sample under test. Unlike other approaches proposed in open literature or employed in the sector, the proposed system can be deployed to provide continuous monitoring of the moisture distribution in silos.

## 1. Introduction

Rice storage is a part of the post-harvest activities and contributes up to 6% losses of harvest [1]. Most of the losses are due to improper storage or moisture build-up due to the surrounding climatic conditions. Moisture content can be defined as the weight of water in the grain mass and expressed in terms of percentage. Based on previous studies [2,3,4,5,6,7,8,9], rice grain moisture content changes with changes in ambient humidity and local weather. Poor aeration could lead to moisture build-up within the bulk of the grain and reduce the quality of the grains. It is therefore important to correctly measure and control the environmental conditions to ensure the quality of the rice in the storage [9]. 

The moisture of rice in a silo can be unevenly distributed. Therefore, the conventional measurement of moisture content can fail depending on the sampling point and it may not represent the moisture content distribution in the silo. Samples taken from a silo do not also accurately represent the moisture within the bulk of the grain. To ensure that the quality of the grain is maintained, thereby reducing losses, it is essential to know the distribution of moisture content in the bulk of the stored grain continually and in real-time, if possible. Different methods had been developed for determining the moisture content of grains [2,3,4,5,6,7,8,9]. However, to the best of the authors’ knowledge, none of the techniques proposes a real-time 3D moisture content measurement of stored rice. 

This paper proposes a new technique that uses radio tomography based on Wi-Fi signals at a frequency of 2.4 GHz. To estimate the location from the Radio Tomography Image (RTI) precisely, the RTI reconstruction is based on noisy measurements [10,11,12]. Conventional RTI methods mostly utilize Tikhonov regularization [13,14] and only consider the correlation properties of the attenuation map. Many available Compressive Sensing (CS) solutions [13], including the least absolute shrinkage and selection operator (LASSO) and orthogonal matching pursuit [3,4], have been proven to successfully reconstruct the image. Under the framework of Bayesian statistics, Bayesian Compressive Sensing (BCS) [15] exploits a priori distribution knowledge of attenuation images to improve the recovery accuracy. However, it requires a reasonable assumption of a prior distribution and it is computationally intensive. It is also reported that the localization accuracy of BCS is less accurate than that of Tikhonov, but the advantage of CS over Tikhonov is that the reconstructed image is cleaner [16]. The Hybrid Tikhonov–LASSO (HTL) combines the advantage of Tikhonov [14] and the LASSO method. 

This paper proposes a novel non-destructive method to determine the moisture content of rice grain using 3D Radio Tomography Imaging (RTI) based on low-cost Wi-Fi signal transmissions and regression-based machine learning approaches. The proposed system can provide continual real-time moisture level distribution in grain stored in a silo. This paper is organized as follows: Section 2 describes the methodology and the experimental setup to measure the moisture contents of rice grain; Section 3 describes the signal processing and the algorithms used to estimate the moisture contents, and Section 4 discusses the results obtained and the conclusions are drawn.

## 2. Methodology

This section presents the four main steps in the experimental measurement. The first step discusses the development of the model test with an analysis of Wi-Fi signals for device-free moisture sensing. The second step assesses the efficiency and accuracy of the localization of moisture content and its distribution based on the RTI method and evaluates the performance of the proposed reconstruction method. Then, a machine learning method is used to predict the moisture values and distribution at unknown levels. The last step shows the process of volume image reconstruction and the volumetric result of the moisture content. 

### 2.1. Experimental Setup

The container for the rice sample used in [14,16], was made of 5 mm thick glass panel with dimensions of 50 cm × 50 cm, and a height of 60 cm to facilitate 2D tomography imaging for localization of moisture distribution in the rice sample. The rice sample filled the container to a height of 50 cm. In this paper, the container has been built to facilitate 3D tomography imaging for the localization of moisture distribution in the rice sample. This will simplify the calculation of the volume of moisture in the sample.

**Figure 1:**
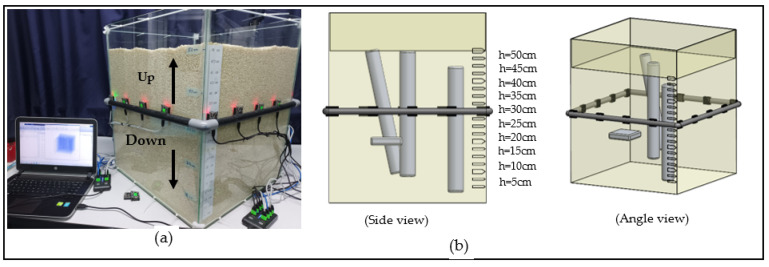
Rice container with the 16 Wi-Fi nodes.

In this experimental setup, 16 Wi-Fi nodes (also known as ESP) were installed on a slider around the sample container where 4 ESP are placed on each side with a distance of 12.5 cm between two nodes. The slider on the outside of the container is made of PVC pipes, which can be slid up and down manually to acquire the Received Signal Strength Indicator (RSSI) [17] data at different levels as shown in Figure 1a. All 16 nodes were used as transceivers. These nodes were connected directly to a PC through USB hubs as shown in Figure 1a. Wi-Fi 2.4 GHz type ESP-12F was used to measure moisture content in rice due to ease of programming and cost-effectiveness. 

To create a 3D tomography image of the moisture content, several tomography images were taken at different levels. The levels indicate the height from the base and the measurements were taken from 5 cm up to 50 cm at intervals of 5 cm. These heights are marked on the container. Four samples with different moisture contents were randomly placed in the rice, as shown in Figure 1b, to simulate areas with different moisture contents from the rest of the grain bulk.

Section 2.2 and Section 2.3 provide detailed information on the number of Wi-Fi nodes used and the process of creating rice samples with different moisture contents.

### 2.2. Number of Nodes

**Figure 2:**
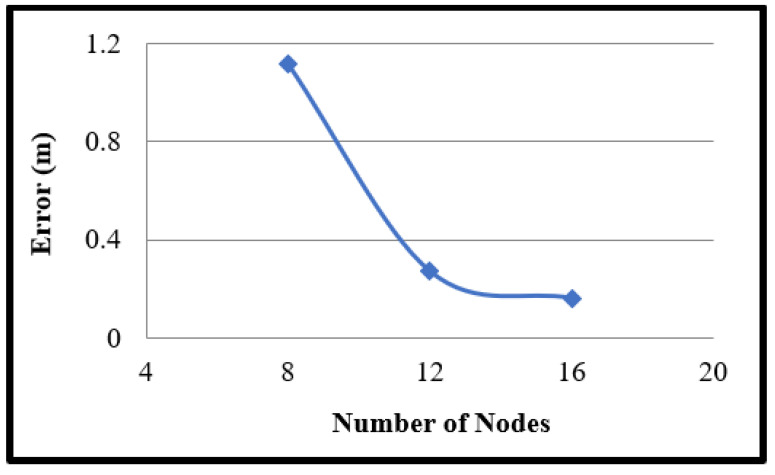
As the number of nodes increases, the error decreases in the same area size.

In this project, the number of Wi-Fi nodes used relied on the average error within the same area. The average error grows as the number of sensors decreases because the link density decreases due to the “blank” spaces between the links. Average moisture content inside the area would not be intersected by enough links if node density is low. As shown in Figure 2, the average error reduces with an increasing number of nodes. Without depending on the transmit power, a maximum distance between two contiguous nodes of 25 cm has been experimentally determined.

The minimum number of nodes required to achieve the best RTI in the same area as reported in previous studies [5,6,7] is given in Equation (1).

(1) $$n=\frac{4\sqrt{a}}{d}\;$$

where,

*n*: number of nodes.

*a*: area.

*d*: distance between two contiguous nodes.

### 2.3. Sample Conditioning

The recommended moisture level for rice storage to maintain optimum quality ranges from 14% to 16% [18]. In this research, the moisture content of rice samples was increased using the moistening method used in the previous research [19,20,21,22]. The samples with different moisture contents can be obtained by adding a predetermined amount of distilled water, Q, as calculated from Equation (2).

(2) $$Q=w_{i}\frac{\left(M_{d}-M_{i}\right)}{\left(100-M_{d}\right)},\;\%\;\mathrm{wb}$$

The Moisture Content of grain is usually determined on a wet basis (wb) [23].

$w_{i}$: Initial mass of the sample in kg.

$M_{i}$: Initial moisture content of sample as % wb.

$M_{d}$: Desired moisture content of the sample in % wb.

Q: Mass of water to be added in kg. 

Using this formula, each bag of the sample with 250 g of rice was moistened. A commercial moisture meter (Meter OGA TA-5) was used to measure the initial moisture content [24]. Once the initial moisture content is measured, the amount of distilled water (*Q*) needed to moisten the samples can be calculated using the formula given in Equation (2). Next, the required amount of distilled water was added to each sample, and the polyethylene bags were resealed. The samples were then stored at a temperature between 4 and 6 °C for 72 h to ensure equal water distribution [25]. Ten hours before the experimental tests were conducted, the samples were taken out of the fridge and kept at room temperature. 

**Figure 3:**
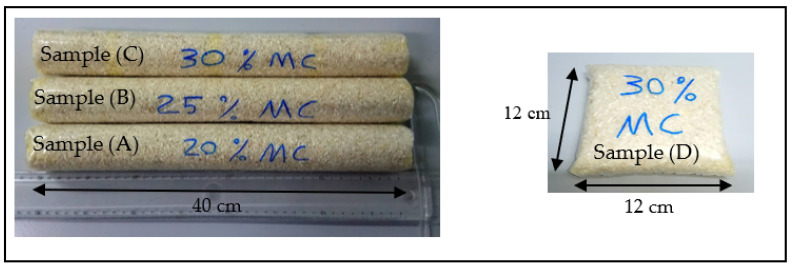
Samples of the rice bags with high moisture contents (MC).

The samples were conditioned in such a way that they can cover all expected moisture contents potential levels. Three samples were put in cylindrical-shaped plastic bags and one sample in a square bag, as shown in Figure 3. Table 1 provides the details of the samples.

## 3. Moisture Content Prediction

### 3.1. Signal Processing

**Figure 4:**
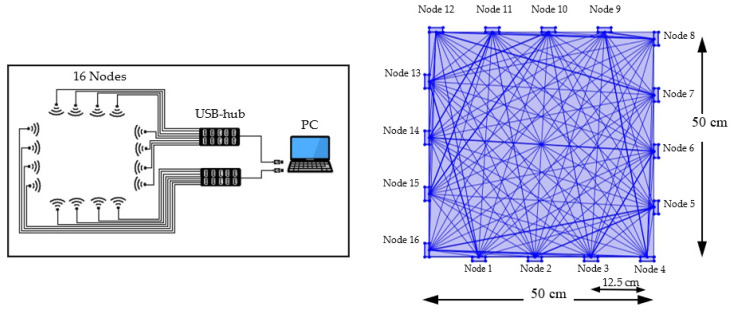
RTI wiring diagram and Wi-Fi mesh Signal illustration.

The experiment was conducted and optimized for a square-shaped grid area and the connectivity between each node is set as a peer-to-peer network. Each node was in the transceiver mode with the transmission power set to 20.5 dB. The model transceiver moisture localization uses the normal moisture content of 14% to relate RSSI measurements with high moisture contents and then estimates the transmitter tag location in a 2D way. For transceiver localization, the RSSI measurements from the wireless links without the test samples, m, were obtained. Assume that *K* is the number of nodes at positions, $\left(x_{k},y_{k}\right)$ *k* = 1, 2, …, *K*, known a priori fixed around the perimeter of the container. In the network, each pair of nodes comprises a link, leading to *L* = *K* (*K* − 1) bidirectional links in total. In addition, each node measures RSSI from another 15 nodes in succession as shown in Figure 4. The RSSI of links will change due to the medium between the nodes, which includes the samples. The impact of the samples on the RSSI often depends on several factors. It is expected that signals, and hence RSSI, propagating through samples with higher moisture contents experience higher levels of attenuation compared to those from samples with lower moisture contents. Due to absorption, RSSI values for signals that have propagated through samples with high moisture contents are smaller and this can be used to determine the location of the sample within the silo. This localization approach is capable of locating multiple moisture areas at the same time [26].

### 3.2. Regularization RTI

The RTI system is used to derive a cross-sectional image vector of an area, based on the power attenuation of the radio signals. In previous research studies, such as in [14], the shadowing-based image vector construction has been used and each radio link is considered where the focus points are located at the sender and receiver nodes. Any high moisture content which lays within the region is considered to be obstructing the corresponding radio link, hence contributing to the signal attenuation. 

Tomography refers to the imaging technique which can be used to evaluate the moisture content by measuring the effect of radio waves passing through the rice grain samples (material-under-test). To obtain the RTI image, the measured Received Signal Strengths (RSS) within the wireless network between the different nodes are used. 

The electric field of signal through lossy materials can be calculated using

(3) $$\left|E\left(z\right)\right|=E_{0}e^{-\alpha z}$$

where E(z) is the electric field at distance z, $E_{0}$, in volt/meter, is the field at a reference point, and α is the attenuation constant. Since power is proportional to the square of the electric field, the power as a function of distance from a reference point, $P_{0},$ can be written as

(4) $$\left|P\left(z\right)\right|=P_{0}e^{-\alpha z}$$

The presence of water affects the relative complex permittivity, ε, of the medium [27]. The relative complex permittivity can be expressed as:

(5) $$\epsilon =\varepsilon^{\prime }+j\varepsilon^{\prime \prime }$$

where $\varepsilon^{\prime }$ is the dielectric constant of the material and $\varepsilon^{\prime \prime }$ is the dielectric loss factor. The relative complex permittivity also depends on the frequency, temperature, bulk density, and composition of the medium. In [27], it has been shown that for plane waves propagating through low loss material, the relative complex permittivity can be calculated as follows:

(6) $$\;\varepsilon^{\prime }={\left(\frac{\beta }{\beta_{0}}\;\right)}^{2},\;\;\varepsilon^{\prime \prime }=\frac{2\alpha \beta }{\beta_{0}^{2}}$$

where $\beta_{0}=\frac{2\pi }{\lambda_{0}}$ is the phase constant and $\lambda_{0}$ is the wavelength of the wave in free space, and $\beta =\frac{\phi }{d}+\beta_{0}$, where ϕ is the phase shift of the propagating wave. $\alpha =\frac{A}{d},$ where A is the attenuation and d is the bulk density [28]. The dependency of the attenuation constant on the dielectric properties of the medium can therefore be expressed as:

(7) $$\propto =\frac{\varepsilon \prime \prime }{2\varepsilon^{\prime }}\left(\frac{\phi }{d}+\frac{2\pi d}{\lambda_{0}}\;\right)$$

Assuming uniform bulk density, d can replace z in Equation (4). Widely used techniques to measure the dielectric constant, $\varepsilon^{\prime }$, and dielectric loss factor, $\varepsilon^{\prime \prime },$ include transmission line (impedance) [29] and free-space (reflection and transmission) [28,30] techniques. Trabelsi et al. have shown that the relative complex permittivity function increases with the moisture content of wheat grain for measurements at a wide range of temperatures and frequencies [27,31,32]. This means that signal attenuation (A) increases in the presence of moisture in grains due to an increase of $\varepsilon^{\prime \prime }$ [27]. Additional losses are due to structural geometric of the components in the medium which affect the absorption and scattering of the propagating signal.

Some researchers have used the RTI concept for different applications [33,34]. If $\Delta y_{i}$ is the resultant difference of RSS value per link, i, and Δx is the RTI image to be reconstructed, n denotes a noise vector, and W is a weighted matrix, $\Delta y_{i}$ can be written as:

(8) $$\Delta \;y_{i}=W\;\Delta x_{i}+n$$

Each of the values is measured in decibels (dB). To simplify, the notations *X* and *Y* are used for Δx and Δy, respectively.

(9) Y=WX+n

For image construction, with Tikhonov regularization [35] applied, can be expressed as:

(10) $$X=(W^{T}W+a\;{\left(D_{x}{}^{T}\;D_{x}+D_{y}{}^{T}\;\;D_{y\;}\right)}^{-1}\;\;W_{y}{}^{T}X$$

In Equation (8), y is the vector of all link difference RSSI measurements, x is the attenuation image that is to be estimated, W is the weight matrix, $D_{x}$ is the difference operator for the horizontal direction, and *D_y_* is the difference operator for the vertical direction, and T is the time of the operator [36].

**Figure 5:**
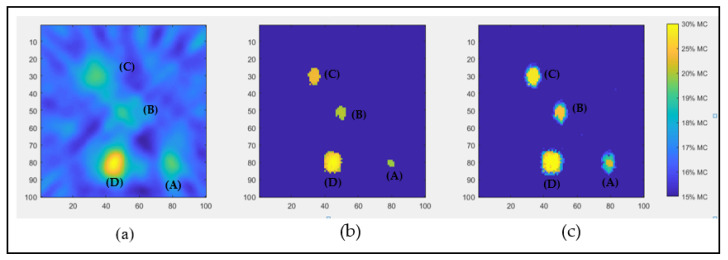
The attenuation image was reconstructed using (**a**) Tikhonov and (**b**) LASSO and (**c**) Hybrid Tikhonov–LASSO (HTL).

For the performances of the RTI reconstruction, two methods, the Tikhonov and LASSO, were used in the grid area on a voxel size of 100 × 100, and the data was read at heights (i.e., 5 cm, 10 cm, 15 cm, 20 cm, 25 cm, 30 cm, 35 cm, 40 cm, 45 cm, and 50 cm). The performance of the solutions based on the Tikhonov regularization, the LASSO method, Hybrid Tikhonov–LASSO (HTL), and a proposed integrated image reconstruction combining Tikhonov and LASSO are presented in this paper. Figure 5 shows the output image of the three reconstruction methods when the high moisture content is located at a height of 20 cm. The Tikhonov regularization method projects blurred images that are corrupted by noise. The sectional shapes of all samples are unclear due to the multiple locations of high moisture content samples. The Tikhonov method is not able to resolve the areas with high moisture contents accurately. By contrast, using the LASSO and HTL, the image is reconstructed with much higher resolutions and is not significantly affected by noise. In addition, the HTL method also provides distinct profiles for the different levels of moisture contents, unlike the other two methods.

Table 2 presents the estimated image quality by Tikhonov, LASSO, and HTL methods. The image quality values are a measure of how accurately the methods estimate the location and dimensions of the samples. The root mean square error (RMSE) values compare the estimated and actual dimensions of the samples. The table shows that the Tikhonov method achieves satisfactory localization accuracy but has the largest imaging error. On the contrary, the LASSO method gives the best image resolution but has a larger localization error than that of the Tikhonov method. Combining the advantages of LASSO and HTL methods, the proposed reconstruction method gets the best localization performance from the HTL method and the image quality of the LASSO method. 

### 3.3. Regression Machine Learning

The regression analysis, which is a machine learning technique, has been used to predict the moisture layers at the different heights, 5–50 cm. In other words, it can be used to predict the moisture values at 7.5 cm, 12.5 cm, 17.5 cm, 22.5 cm, 27.5 cm, 32.5 cm, 37.5 cm, 42.5 cm and 47.5 cm using the datasets at the known heights (i.e., 5 cm, 10 cm, 15 cm, 20 cm, 25 cm, 30 cm, 35 cm, 40 cm, 45 cm, and 50 cm).

**Figure 6:**
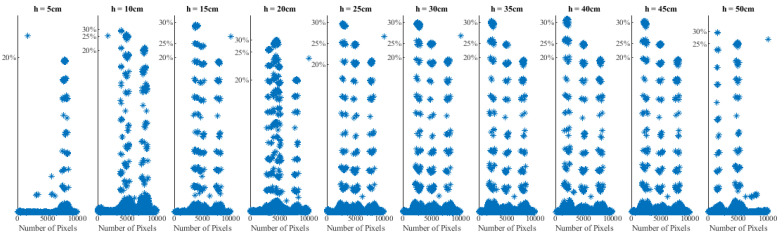
Scatter plot for regression machine learning.

The regression analysis uses the given information to provide the best-fit equations for the layers (heights) in the form expressed in Equation (11). Figure 6 shows the scatter plots of results of moisture contents prediction using a regression model for measurements at different heights (10 cm to 50 cm). It shows how the accurate detection of samples varies with measurement location. It should be noted that accuracy is reduced at the bottom due to the effect of signal reflection and scattering from the table surface. This can be minimized by placing the container on raised legs. The regression formulae were obtained from these scatter plots using RMSE values to determine the best-fit equations:

(11) $$y=a+bx+cx^{2}+dx^{3}+ex^{4}\;$$

where *x* is layer height; *y* is moisture value; and *a*, *b*, *c*, *d*, and *e* are equation parameters. 

**Figure 7:**
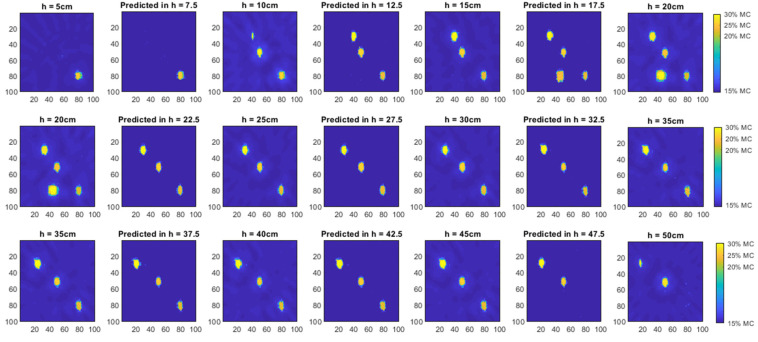
The locations of moisture distribution in (5 cm, 10 cm, 15 cm, 20 cm, 25 cm, 30 cm, 35 cm, 40 cm, 45 cm, and 50 cm) and predicted in high (7.5 cm, 12.5 cm, 17.5 cm, 22.5 cm, 27.5 cm, 32.5 cm, 37.5 cm, 42.5 cm and 47.5 cm).

In this case, the data is the given moisture values at 10 intervals. This data is a 100 × 100 matrix which is to be converted into a vector of 10,000 values. Then, the regression technique is used to create 10,000 equations (each for every pixel location). The parameters for each equation are calculated at heights of 7.5, 12.5, 17.5, 22.5, 27.5, 32.5, 37.5, 42.5, and 47.5, and the moisture content values are also calculated. The values are converted from a vector to a matrix and then saved in layers. Figure 7 shows the locations of moisture distribution at the top layer.

After estimating the level of moisture at unknown layers, the displacement of the points of moisture that corresponds to that of the moisture content is found and tracked. For a fair evaluation of the performance of the external RTI method reported in this work and comparison with previous work, a blob detection technique with multiple moisture tracking has been applied. This has been used in many studies such as objective tracking and localization in robotics [26].

### 3.4. Volumetric Moisture Content

Volumetric moisture content images, such as computed tomography (CT) scans, which have been used in the medical field [24,25,26], consist of a series of stacked two-dimensional (2D) images that allow for a more accurate representation of the three-dimensional (3D) moisture distribution [37,38,39,40]. The aim of this is an indirect configuration of the internal moisture distribution locations in a three-dimensional manner, which helps to calculate the volumetric moisture content in the rice.

**Figure 8:**
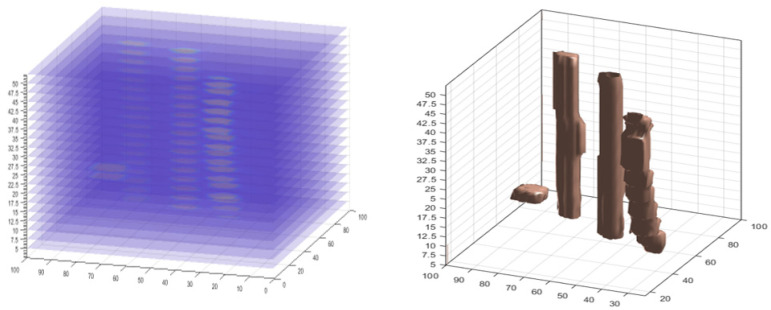
Stacking 2D slices to create a 3D model.

In recent years, there has been a steady increase in the use of computed tomographic images, such as the analysis of the internal structures of materials [33,41]. Other researchers have used it to analyze the properties of the soil [34,42]. For this project, the 19 computed tomography images have been used with a size of 100 × 100 pixels, 2.5 cm between each sliced RTI, as shown in Figure 8. According to the 3D graph, high moisture rice samples were found and can be localized clearly.

Volumetric moisture content data consist of slices of RTI’s x, y, z, and v matrices as three-dimensional matrices, where x, y are the coordinates of pixels in the plane, z is the height of each slice, and v is the actual image slices, possibly as pixel density values.

Surface rendering was originally applied to volumetric data to present a more direct method for 3D visualization of moisture distribution from 2D slices [10]. This made it possible to compare the volume of the actual four rice samples with the size results for 3D model, as shown in Table 3.

## 4. Conclusions

This paper presented some improvements to the quality and imaging performance of RTI in challenging rice-filled environments. Due to the measured area, the RSS differences caused by differences in rice moisture contents can be detected and located within the rice bulk. The RTI improvement quality was applied to build a novel concept in the form of a non-destructive method for localizing moisture distribution sensing in stored grains (Rice) in real-time. It was demonstrated that existing accuracy improvement methods are completely depending on the number of Wi-Fi transceiver nodes and the sample volume. Rice samples with 20% moisture content, which is closer to the baseline moisture contents of 14%, were successfully detected and localized, but with a lower image quality. 

The higher the number of wireless nodes used, the higher the computational power required to process the data. In this study, 16 nodes were sufficient for detecting and localizing different moisture contents of rice in a silo, although the number of nodes will depend on the dimensions of the silo. The results in this paper have demonstrated a promising methodology that offers good accuracy. Two major contributions to this are: (1) the development of moisture detection and variance within stored grains which could be conducted continually in real-time using low-cost RF systems; (2) using a tomographic technique to detect and localize moisture contents variation within grain bulk (volumetric system), with accuracies between 83.7% to 93.4%. The technique and algorithm presented in this paper can be used to provide real-time continuous monitoring of moisture hotspots within grain storage systems [43]. The number of nodes and size of the silo can be adapted accordingly. 

## Figures and Tables

**Table 1:** Details of the physical sizes of the samples.

	Sample (A) 20% MC	Sample (B) 25% MC	Sample (C) 30% MC	Sample (D) 30% MC
Size (cm)	length = 40 diameter = 5	length = 40 diameter = 5	length = 40 diameter = 5	length = 12 width = 12 thickness = 2
Weigh (kg)	0.65	0.65	0.65	0.45
Added water (kg)	0.04	0.086	0.139	0.096

**Table 2:** Performance evaluation for Tikhonov, LASSO, and HTL.

	Tikhonov	LASSO	(HTL)
Image Quality	27%	66%	93%
RMSE	0.14	0.12	0.08

**Table 3:** The ratio of the size of the volume between Real Volume and 3D Volume.

	Sample (A) with 20% MC	Sample (B) with 25% MC	Sample (C) with 30% MC	Sample (D) with 30% MC
Real Volume (cm^3^)	785	785	785	288
3D Volume (cm^3^)	711.03	729.131	732.87	241
Size Quality (%)	90.57707	92.88293	93.35924	83.68056

## Data Availability

All data generated or that appeared in this study are available upon request by contact with the corresponding author. Furthermore, the models and code used during the study cannot be shared at this time as the data also form part of an ongoing study.
